# Characteristics of C4-Type Zinc Finger Proteins in Tardigrades and Their Responses in Extreme Environments

**DOI:** 10.3390/ijms27041739

**Published:** 2026-02-11

**Authors:** Mingwang Duan, Yukai Zhou, Zhaoyi Li, Xiaojing Shi, Zefeng Hua, Zhidan Gao, Dong Yang

**Affiliations:** 1 State Key Laboratory of Medical Proteomics, National Center for Protein Sciences, Beijing 102206, China; 2 College of Life Science, Hebei University, Baoding 071002, China; 3 Disease Prevention and Control Department, Characteristic Medical Center of PLA Rocket Force, Beijing 100088, China

**Keywords:** tardigrades, C4-type zinc finger proteins, gene families, desiccation, radiation

## Abstract

The regulatory role of zinc finger proteins is crucial for the development of extreme environmental tolerance in numerous species. Tardigrades, as multicellular animals capable of withstanding multiple extreme conditions, have yet to undergo systematic identification and analysis of their zinc finger proteins. This study first analysed the distribution characteristics of zinc finger proteins across four species of tardigrades. We systematically characterised the family features of C4-type ZFPs in these four species and explored their regulatory roles in extreme environmental adaptation. Statistical genomic investigation reveals a wide distribution of the C4-type ZFP family among tardigrades. Phylogenetically, they separate into six distinct groups, with Group 5 emerging as a functionally specialised branch enriched in stress-responsive promoter elements. Molecular evolutionary evidence points to tandem duplication as key to this branch’s expansion and functional innovation, while also highlighting the pervasive purifying selection across this family. Transcriptomic analysis revealed that a specific subset of C4-type ZFP genes belonging to phylogenetic Group 5 in both *H. henanensis* and *H. exemplaris* were downregulated after irradiation. Functional enrichment indicates that these genes are linked to nuclear receptor transcription factor activity and the negative regulation of NF-κB signalling. We propose that their coordinated downregulation may represent a conserved stress-adaptive response, potentially derepressing NF-κB and reprioritizing resources toward damage repair over growth. This study provides a foundation and key clues for research aimed at elucidating the functional mechanisms of C4-type ZFPs in tardigrade extremotolerance.

## 1. Introduction

Zinc finger domains constitute a class of finger-like structural motifs. Initially identified as DNA-binding motifs within the *Xenopus laevis* transcription factor TFIIIA [1], they are now recognised for their capacity to bind DNA, RNA, proteins, and/or lipid substrates [2]. Proteins containing zinc finger domains are termed zinc finger proteins, which play a crucial role in gene expression regulation. They participate in diverse functions including gene transcription, translation, mRNA transport, cytoskeletal organisation, epithelial development, cell adhesion, protein folding, chromatin remodelling, and zinc sensing [3]. Zinc finger proteins can be classified into multiple types such as C2H2, C4, and C6 based on their conserved motifs [4,5]. Previous studies have revealed that zinc finger proteins are closely associated with physiological and metabolic processes in plants responding to various abiotic stresses [6,7]. Among these, CCCH-type zinc finger proteins may contribute to barley’s resistance to cold stress, metal toxicity, and salt stress [8], while Q-type C2H2 zinc finger proteins participate in grapevine’s responses to exogenous hormones and low-temperature tolerance [9].

Tardigrades, also known as water bears, belong to the phylum Tardigrada within the superphylum Panarthropoda, exhibiting extraordinary radiation tolerance to multiple radiation types [10]. Some terrestrial tardigrade species also utilise desiccation cryptobiosis to withstand adverse environments [11]. Recent studies indicate that multiple proteins play crucial roles in tardigrade radiation resistance and desiccation tolerance, including Dsup proteins and tardigrade heat-soluble proteins [12,13,14]. However, systematic research on the distribution and features of zinc finger proteins in tardigrades and their role in responding to extreme environmental conditions remains lacking. In this study, four tardigrade species with available well-annotated genomes were selected: *Hypsibius henanensis* (cultivated in our laboratory), *Hypsibius exemplaris*, *Ramazzottius varieornatus*, and *Paramacrobiotus metropolitanus*. Our analysis focused on the distribution of zinc finger domains within tardigrades, the characteristics of C4-type ZFPs, and their responses under extreme environmental conditions.

## 2. Results

### 2.1. Identification, Classification and Characteristic Analysis of the C4-Type ZFP Family in Tardigrades

**Figure 1:**
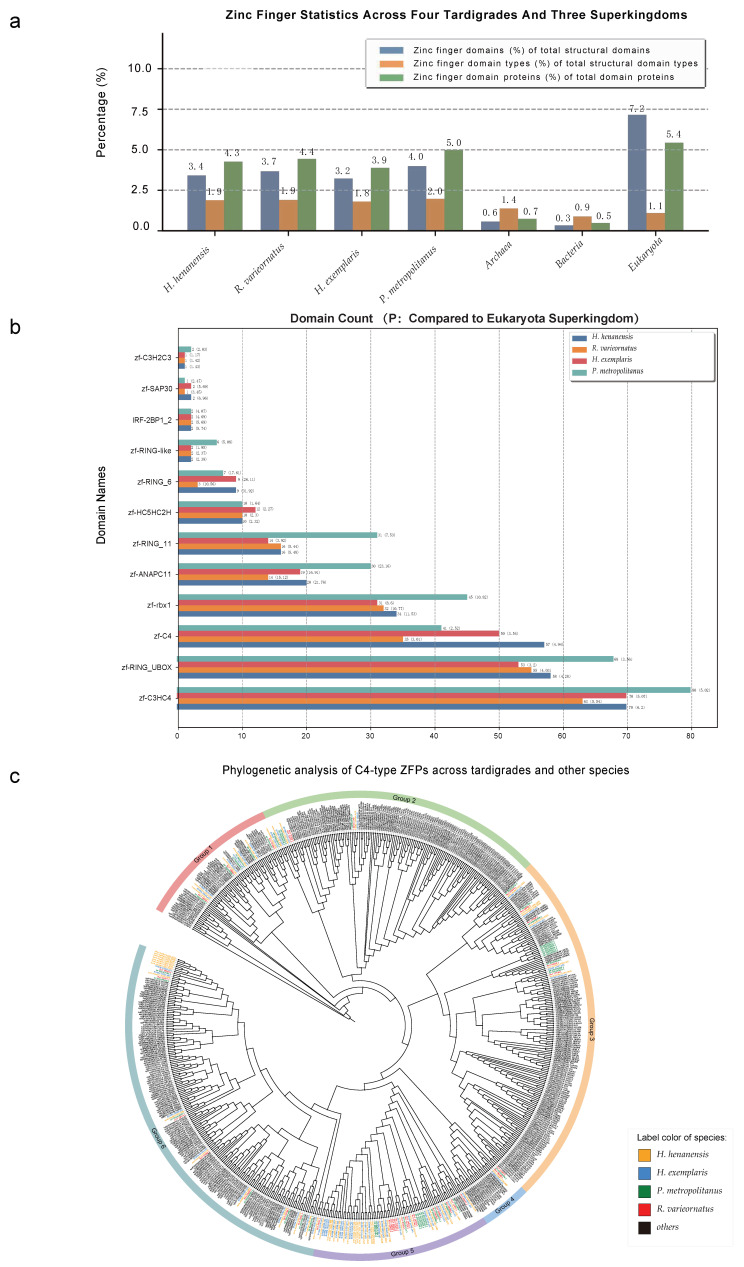
The overall distribution of zinc finger domains and phylogenetic analysis of C4-type ZFPs: (**a**) The overall distribution of zinc finger domains across four tardigrade species and three superkingdoms; (**b**) the count of zinc finger domains in four tardigrade species and the relative ratio *p* compared to the Eukaryota superkingdom; (**c**) phylogenetic analysis of C4-type ZFPs in four tardigrade species and other species.

The genes and their corresponding protein sequences from four tardigrade species were collected. Following the principle that each gene is represented by its longest protein sequence, the distribution of zinc finger domains was statistically analysed across *H. henanensis*, *H. exemplaris*, *R. varieornatus*, and *P. metropolitanus*. Additionally, we selected 1874 representative species from the superkingdoms Archaea, Bacteria, and Eukaryota, and statistically analysed the distribution of zinc finger domains (Figure 1a and Table S1). The ratio of the number of zinc finger domains of each type to the total number of domain proteins was then used as a measure of zinc finger domain distribution. The ratio m was calculated for each type of zinc finger domain relative to the total number of domain proteins in the four tardigrades, and the ratio M was calculated for each type of zinc finger domain relative to the total number of domain proteins across the three superkingdoms. The relative ratio *p* was calculated as m/M, to provide a rough comparison of the relative abundance of zinc finger domains across the four tardigrades versus the three superkingdoms. Within the defined species scope, twelve zinc finger domains were found to exist exclusively in Eukaryota: zf-C3HC4 (PF00097), zf-C4 (PF00105), zf-RING-like (PF08746), IRF-2BP1_2 (PF11261), zf-rbx1 (PF12678), zf-ANAPC11 (PF12861), zf-RING_UBOX (PF13445), zf-HC5HC2H (PF13771), zf-SAP30 (PF13866), zf-RING_6 (PF14835), zf-C3H2C3 (PF17122), and zf-RING_11 (PF17123). In addition, these twelve zinc finger domains exhibited relatively higher distribution across the four tardigrade species compared to the eukaryotic domain (*p* > 1). Furthermore, zf-C3HC4, zf-RING_UBOX, and zf-C4 ranked among the top three in abundance within the four tardigrade species compared to other zinc finger domain types (Figure 1b and Table S2). Following collection of raw transcriptomic data for *H. exemplaris* and *R. varieornatus* under extreme radiation and desiccation conditions [12,15,16,17], and for *P. metropolitanus* under extreme desiccation conditions [18], differential expression analysis (Table S3) and domain annotation of differentially expressed molecules revealed that among these 12 zinc finger domains, differentially expressed genes common to all three tardigrades belonged to the zf-C3HC4, zf-RING_UBOX, and zf-C4 types (Table S4). Notably, zf-C4 serves as the DNA-binding domain for nuclear hormone receptors in nearly all cases. DNA-binding domains exert activating or repressive effects by binding specific regions on DNA termed hormone response elements. They also participate in diverse functions including nuclear localisation and interactions with transcription factors and co-activators [19,20]. To investigate gene expression regulation mechanisms in tardigrades under extreme conditions, proteins containing the zf-C4 domain were selected as the primary focus of this study.

Based on homology and hidden Markov models, 57, 50, 36, and 42 C4-type ZFPs were identified in *H. henanensis*, *H. exemplaris*, *R. varieornatus*, and *P. metropolitanus* respectively. A systematic analysis was conducted on the coding sequence (CDS) length, protein length, molecular weight, instability index, and hydrophobicity of the C4-type ZFPs (Table S5). It was found that almost all of them (184 proteins) were hydrophilic, with the exception of only one protein (RvY_06339.1) from *R. varieornatus*. Furthermore, the secondary structures of C4-type ZFPs in the four tardigrades were predominantly composed of α-helices, extended chains, and disordered coils.

A phylogenetic tree was constructed using 185 C4-type ZFPs identified in four species of tardigrades alongside high-quality, manually annotated C4-type ZFPs from other species in the Swiss-Prot database. Following manual rooting with a C4-type ZFP from the purple sea urchin (*Strongylocentrotus purpuratus*) as an outgroup, the C4-type ZFPs were grouped into six distinct clades based on their phylogenetic relationships (Figure 1c). The clustering results with other species’ high-quality C4-type ZFPs manually annotated in Swiss-Prot further validate the accuracy of identifying C4-type ZFPs in the four tardigrade species. Phylogenetic tree analysis revealed that C4-type ZFPs from each tardigrade species were scattered across each group, while Group 5 comprised almost exclusively C4-type ZFPs from tardigrades.

### 2.2. Analysis of Conserved Motifs in C4-Type ZFPs from Four Tardigrade Species

**Figure 2:**
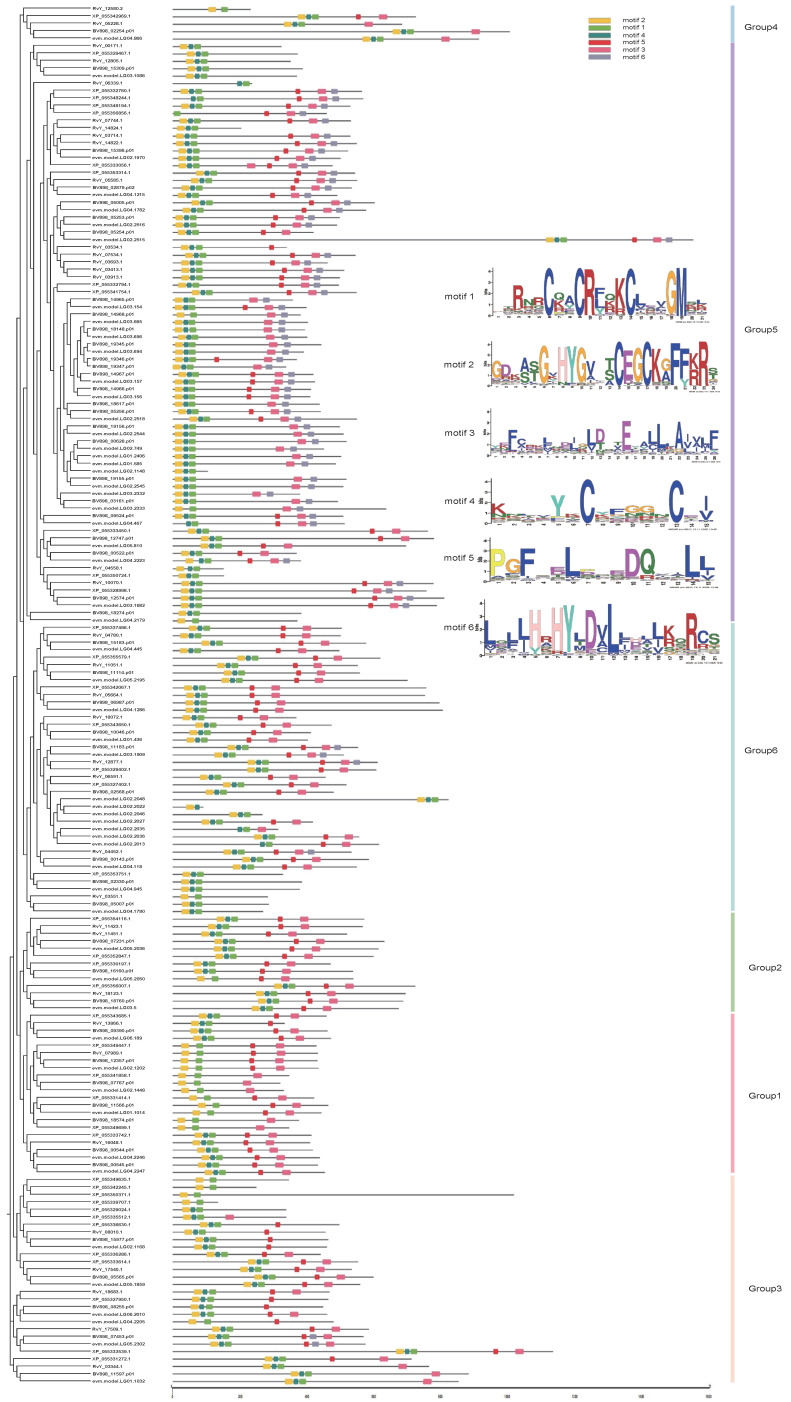
Phylogenetic relationships and architecture of conserved protein motifs in C4-type ZFPs of *H. henanensis*, *H. exemplaris*, *R. varieornatus*, and *P. metropolitanus*. The phylogenetic tree was constructed based on the protein sequences of C4-type ZFPs using FastTree 2.1.11 in Lg+Gamma mode. The motif compositions of ZFP members, numbered 1–6, are displayed in different-coloured boxes.

Using MEME Suite 5.5.9, a total of six conserved amino acid sequence motifs were analysed for C4-type ZFPs from four tardigrade species (Figure 2 and Table S6). InterPro database searches indicated that among the six motifs, motif 1 and motif 2 both represent C4-type zinc finger structures. Motif 1 (RTRNRCQACRFQKCLEVGMSL) constitutes a classic nuclear hormone receptor DNA-binding domain, capable of recognising specific genomic DNA sequences to anchor proteins at precise locations. Reactome [21] pathway analysis indicates that proteins containing this domain participate in an exceptionally broad range of hormone signalling pathways [19,20], including but not limited to: steroid hormone signalling, thyroid hormone signalling, retinoic acid (vitamin A derivative) signalling, and vitamin D metabolism and signalling. These annotations are highly abundant across multiple species including humans, mice, and rats, suggesting that this function is highly conserved evolutionarily. Motif 2 (GDKATGVHYGVLTCEGCKGFFKRS) constitutes the DNA-binding domain of nuclear hormone receptors belonging to the NR3 subfamily [22,23,24]. This domain is responsible for sequence-specific DNA recognition and binding, serving as the core component enabling nuclear hormone receptors to function as transcription factors. It recognises specific hormone response elements, enabling the corresponding receptor protein to activate or repress downstream gene expression upon receiving the relevant hormone signal. This domain plays a central role in physiological processes such as stress responses, metabolism, development, and reproduction. Proteins containing this domain are extensively involved in steroid hormone signalling pathways, including gene expression regulatory networks associated with glucocorticoids and sex hormones. Motif 3 (YEFCRKLNDJGLDQTEYALLLAIVLF) constitutes the nuclear hormone receptor ligand-binding domain, primarily responsible for: ligands (e.g., steroids, thyroid hormones, vitamin A/D derivatives), mediating dimerisation (forming dimers with another nuclear hormone receptor), and recruiting co-factors (post ligand binding, its conformation changes to recruit co-activator or co-repressor complexes, ultimately determining whether target genes are activated or repressed) [25,26,27]. Motif 5 (PGFSELPQEDQLALL) similarly constitutes the ligand-binding domain of nuclear hormone receptors, responsible for hormone binding and regulation of transcriptional activity. Overall, motif 1 and motif 2, as the DNA-binding domains of nuclear hormone receptors, exhibit high conservation across nearly all phylogenetic members, absent only in a very small number of cases. Furthermore, members within the same phylogenetic branch exhibit minor variations, while those across different branches show significant differences in motif types and positions. Compared to other groups, a large number of members in Group 5 contain all motif types: both motif 1 and motif 2 of the nuclear hormone receptor DNA-binding domain, and motif 3 and motif 5 from the nuclear hormone receptor ligand-binding domain, as well as functionally uncharacterised motif 4 (KNLVYKCYFGGNCEI) and motif 6 (LQLJHRHYLDVLLDLLKQRCS).

### 2.3. Cis-Acting Element Analysis of C4-Type ZFP Genes in Four Tardigrade Species

**Figure 3:**
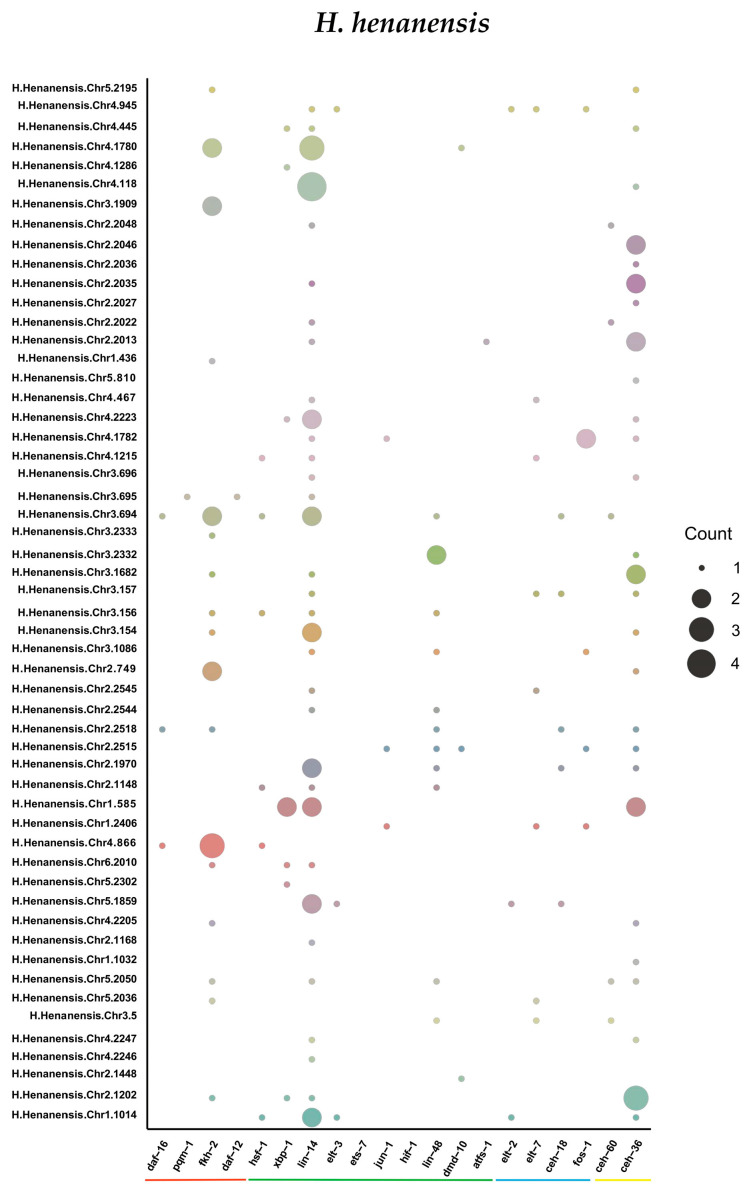
Distribution of major stress and hormone response-related cis-acting regulatory elements in the promoters of *H. henanensis*’s C4-type ZFP genes. Note: Different colours of the circles represent different genes, and the size of a circle represents the number of corresponding cis-elements. The four colours below the X-axis label divide the labels into four categories: red for stress signal integration, green for specific environmental stress response, blue for pathogen immunity, and yellow for collaborative regulation.

Transcription factor binding sites from the nematode CORE dataset within the JASPAR2024 database [28] were used to predict cis-acting elements for C4-type ZFP genes of the four tardigrade species. As a result, twenty cis-acting elements associated with stress signal integration, specific environmental stress responses, pathogen immune responses, and auxiliary/coregulatory mechanisms were identified within the 2000 bp sequence upstream of the ATG start codon: daf-16, pqm-1, fkh-2, daf-12, hsf-1, xbp-1, lin-14, elt-3, ets-7, jun-1, hif-1, lin-48, dmd-10, atfs-1, elt-2, elt-7, ceh-18, fos-1, ceh-60, and ceh-36, corresponding to the motifs ‘GTAAACAC, ACTGATAA, [AG]TAAACA, G[AT]G[CT]G[CT]GTGTG[CT]GT, TTC[CT]AGAA, [AT]TACGTG, GAAC[AG]C, TCTTATCA, AACCGGAAGT, ATGACTCA, T[CT]ACGTGAC, CCGTTA[CT], AATGTTTC, ATGATGCAA[AT], TCTTATCA, TGATAAC, TGCATA[AT]T, TGACTCA, TGATTGACA, GGATTA’. Among these, 18, 15, 10, and 17 motifs were identified in *H. henanensis*, *H. exemplaris*, *R. varieornatus*, and *P. metropolitanus*, respectively (Figure 3 and Figures S1–S3, and Table S7). Notably, eight motifs (pqm-1, fkh-2, hsf-1, xbp-1, lin-14, lin-48, elt-7, ceh-36) were found in all four tardigrade species. Moreover, the prediction results indicate that these cis-acting elements are widely present in the C4-type ZFP genes of tardigrades, occurring in 174 out of 185 C4-type ZFP genes.

### 2.4. Analysis of the Evolutionary Characteristics of C4-Type ZFP Genes in Four Tardigrade Species

**Figure 4:**
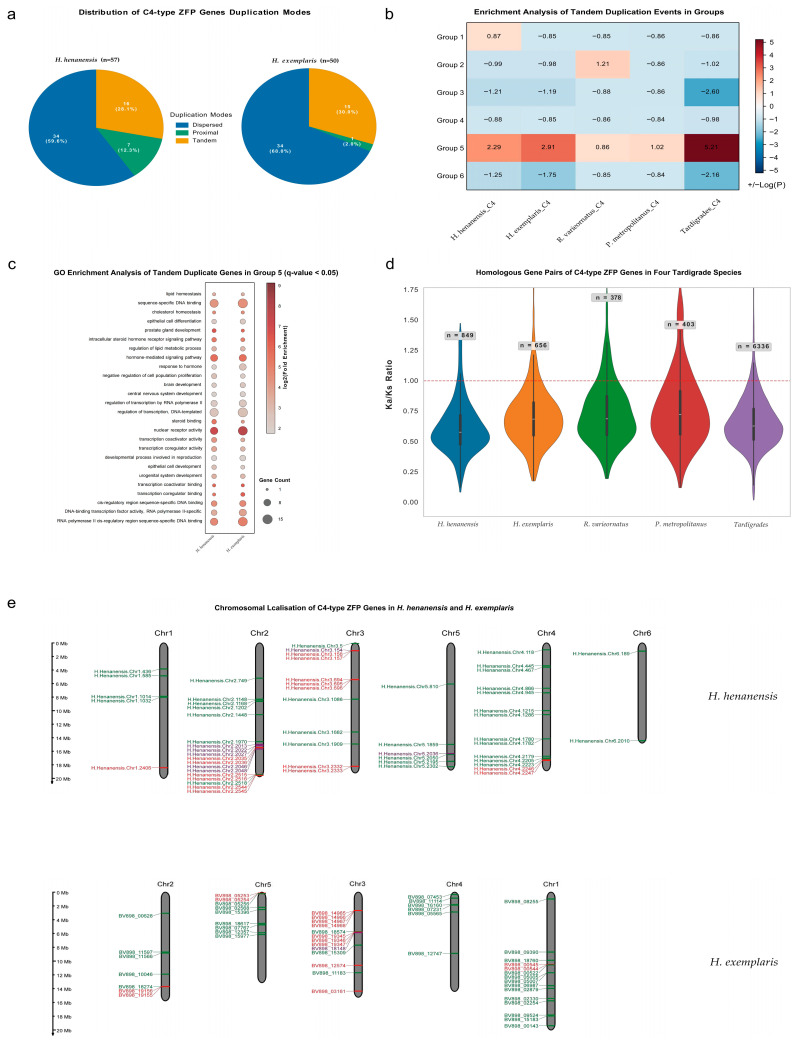
Analysis of the evolutionary characteristics of C4-type ZFP genes in four tardigrade species. (**a**) Distribution of C4-type ZFP gene duplication modes in *H. henanensis* and *H. exemplaris*; different gene duplication modes are represented by different colours, and the white numbers represent the corresponding number and proportion of C4-type genes. (**b**) Enrichment analysis of tandem duplication events in Group 5 C4-type ZFP genes of tardigrades relative to those across the entire C4-type ZFP gene family of tardigrades. (**c**) GO enrichment analysis of tandem duplicate genes in Group 5 (q-value < 0.05); the depth of bubble colour indicates the enrichment fold, while bubble size represents the number of genes enriched for the corresponding functional category. (**d**) The Ka/Ks ratio of homologous gene pairs of C4-type ZFP genes in four tardigrade species; n represents the number of homologous gene pairs. (**e**) Chromosomal localisation of C4-type ZFP genes in the tardigrades *H. henanensis* and *H. exemplaris*. Different label colours denote distinct duplication types: red signifies tandem duplicates, purple denotes proximal duplicates, and green represents dispersed duplicates. Note: *H. henanensis*’s Chr1, Chr2, Chr3, and Chr5 exhibit collinear relationships with *H. exemplaris*’s Chr2, Chr5, Chr3, and Chr4 respectively. Furthermore, *H. henanensis*’s Chr4 and 6 are collinear with *H. exemplaris*’s Chr1.

Analysis of gene duplication events revealed that dispersed duplication was the dominant mode for C4-type ZFP gene amplification in *H. henanensis* and *H. exemplaris*, while tandem and proximal duplication played secondary, yet contributory, roles in the expansion of these genes. Specifically, among the 57 C4-type ZFP genes in *H. henanensis*, 34 were dispersed duplicates, 16 were tandem duplicates, and 7 were proximal duplicates. Among the 50 C4-type ZFP genes in *H. exemplaris*, 34 were dispersed duplicates, 15 were tandem duplicates, and 1 was a proximal duplicate (Figure 4a). Furthermore, in *R. varieornatus* and *P. metropolitanus*, which lack chromosome-level genome assemblies, tandem and proximal duplications of C4-type ZFP genes are less common (Figure S4). Enrichment analysis conducted upon gene duplication events revealed that tandem duplications within C4-type ZFP genes of the three tardigrade species (*H. henanensis*, *H. exemplaris*, *R. varieornatus*) were significantly over-represented relative to comparable events across their entire genomes (Table S8). This indicates that tandem duplication might drive the expansion of C4-type ZFP genes in the tardigrades *H. henanensis*, *H. exemplaris*, and *R. varieornatus*. Notably, many of these tandemly duplicated C4-type ZFP genes belong to Group 5. Further enrichment analysis revealed that, within the phylogenetic tree, tandem duplication events occurring in Group 5 C4-type ZFP genes of tardigrades were significantly over-represented relative to those occurring across the entire C4-type ZFP gene family. This indicates that tandem duplication events played a crucial role in the expansion of Group 5 within the C4-type ZFP gene family of tardigrades across the phylogenetic tree (Figure 4b). GO enrichment analysis of tandemly duplicated C4-type ZFP genes in Group 5 from *H. henanensis* and *H. exemplaris* separately revealed that their functions are mainly related to transcriptional regulation, nuclear receptor and hormone signalling, development and differentiation processes, metabolism and homeostasis regulation, cell proliferation and death regulation, etc. These entries were absent in genes outside the tandemly duplicated C4-type ZFP genes within Group 5 (Figure 4c).

To investigate the characteristics of gene age distribution in C4-type ZFP genes of tardigrades, gene ages within the tardigrade genome were determined based on protein sequence homology across species. Results indicate that among the 185 C4-type ZFPs, the majority (173) were categorised into the Eukaryota gene age grade. The remaining 12 proteins fell into other grades, with two classified into the Cellular origanisms-grade, eight as Metazoa-grade, and two as tardigrade-specific (Table S9).

Based on orthologous and paralogous gene pairs, interspecific and intraspecific Ka/Ks analyses of C4-type ZFP genes in four tardigrade species (Figure 4d and Table S10) revealed that interspecifically, among 6336 orthologous gene pairs of C4-type ZFP genes across the four tardigrade species, 355 exhibited a Ka/Ks ratio greater than 1, while 5981 had a ratio less than 1. Over 94% of gene pairs displayed a Ka/Ks ratio below 1, indicating that C4-type ZFP genes in these four tardigrades undergo primarily purifying selection during interspecific evolution, with functional conservation prevailing. With fewer than 6% of gene pairs exhibiting a Ka/Ks ratio above 1, this suggests that functional differentiation may be driven by positive selection. Intraspecifically, analysis of paralogous C4-type ZFP gene pairs revealed a clear pattern of purifying selection across all four tardigrade species. The vast majority of gene pairs exhibited Ka/Ks ratios below 1 (*H. henanensis*: >94% of 656 pairs; *H. exemplaris*: >90% of 849 pairs; *R. varieornatus*: >87% of 403 pairs; *P. metropolitanus*: >82% of 378 pairs), while only a minor fraction showed ratios exceeding 1. The above findings indicate that homologous gene pairs of C4-type ZFP genes in the four tardigrade species exhibit consistent evolutionary patterns both within and between species, primarily undergoing purifying selection, with a minority of homologous gene pairs experiencing positive selection.

Chromosomal localisation analysis of C4-type ZFP genes revealed that in the tardigrades *H. henanensis* and *H. exemplaris*, these genes exhibit uneven chromosomal distribution yet show relative concentration in regions of tandem duplication and proximal duplication. The 57 and 50 C4-type ZFP genes in *H. henanensis* and *H. exemplaris* are distributed unevenly across six (Chr1-6) and five (Chr1-5) chromosomes, respectively (Figure 4e). Genes encoding C4-type ZFPs arising from local duplication comprise 16 in *H. henanensis*, predominantly concentrated on chromosomes 2 and 3, and 15 in *H. exemplaris*, primarily clustered on chromosome 3. Previous studies from our laboratory [29] indicate that *H. henanensis*’s Chr1, Chr2, Chr3, and Chr5 exhibit collinear relationships with *H. exemplaris*’s Chr2, Chr5, Chr3, and Chr4 respectively. Furthermore, *H. henanensis*’s Chr4 and 6 are collinear with *H. exemplaris*’s Chr1. In addition, it can be seen that all locally replicated C4-type ZFP genes in *H. henanensis* and *H. exemplaris* have homologs derived from duplication events, with the sole exception of the BV898_12574 gene on Chr3 in *H. exemplaris* (Table S11).

### 2.5. Subcellular Localisation of C4-Type ZFPs

**Figure 5:**
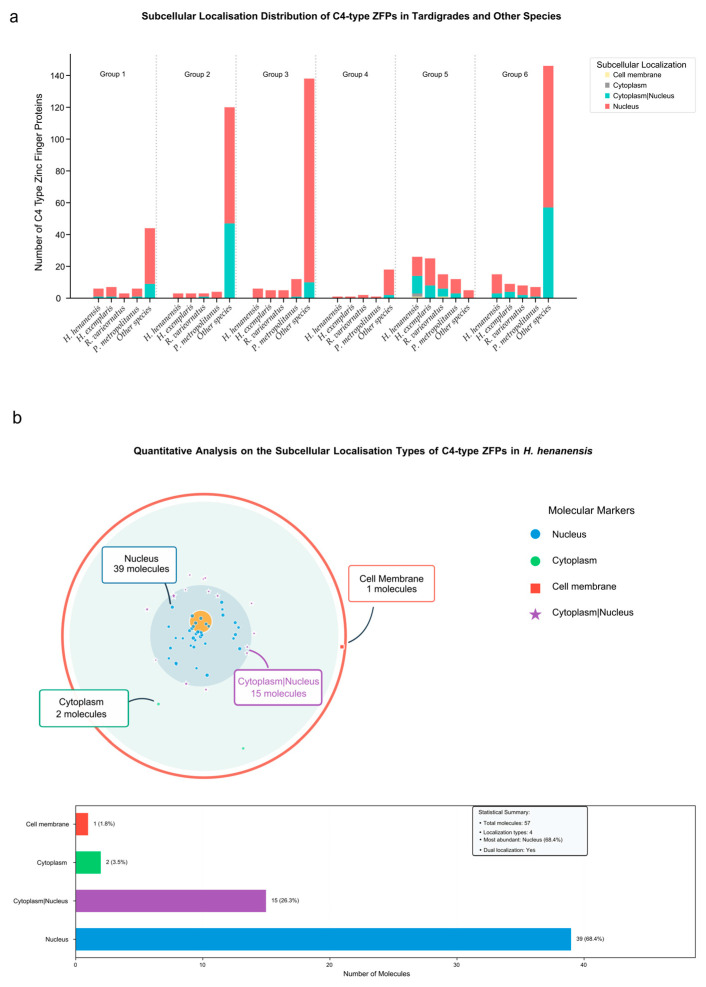
The predicted subcellular localisation of C4-type ZFPs. (**a**) Statistical cumulative frequency plot of predicted subcellular localisation for C4-type ZFPs across four tardigrade species and other species within the phylogenetic tree; (**b**) quantitative analysis on the subcellular localisation types of C4-type ZFPs in *H. henanensis*.

Subcellular localisation prediction was performed using a combined approach with TargetP-2.0 [30] and DeepLoc 2.1 [31]. The predicted subcellular localisation of C4-type ZFPs within the phylogenetic tree revealed relatively conserved patterns across tardigrade species (Figure 5 and Figures S5–S8, and Table S12). Specifically, the C4-type zinc finger proteins in each group of tardigrades are primarily localised in the nucleus, followed by ‘cytoplasm|nucleus’ (dual localization of cytoplasm and nucleus). This is a consistent feature across Groups 1, 2, 3, 5, and 6.

Notably, Group 5 exhibits a distinct and diverse localisation pattern. Besides the nucleus and ‘cytoplasm|nucleus’, a few members in Group 5 are present in the cytoplasm and cell membrane. Furthermore, among the 185 C4-type ZFPs in tardigrades, 42 members are not only localised in the nucleus, and members not only localised in the nucleus exist in all four tardigrade species. Among these 42 members, 27 are found in Group 5, which is a considerable proportion when compared to other groups.

Similarly, among C4-type ZFPs in other species, 125 members are not only localised within the nucleus. Of these 125 members, 48 originate from *Caenorhabditis elegans* and are predominantly found in Group 2.

### 2.6. Differential Expression and Functional Analysis of C4-Type ZFP Genes in Tardigrades Under Extreme Conditions

To further investigate the response mechanisms of tardigrade C4-type ZFP genes to extreme conditions, we performed differential expression analysis of these genes using publicly available transcriptomic data from *H. exemplaris*, *H. henanensis*, *R. varieornatus*, and *P. metropolitanus* subjected to radiation or desiccation [12,15,16,17,18,29].

**Figure 6:**
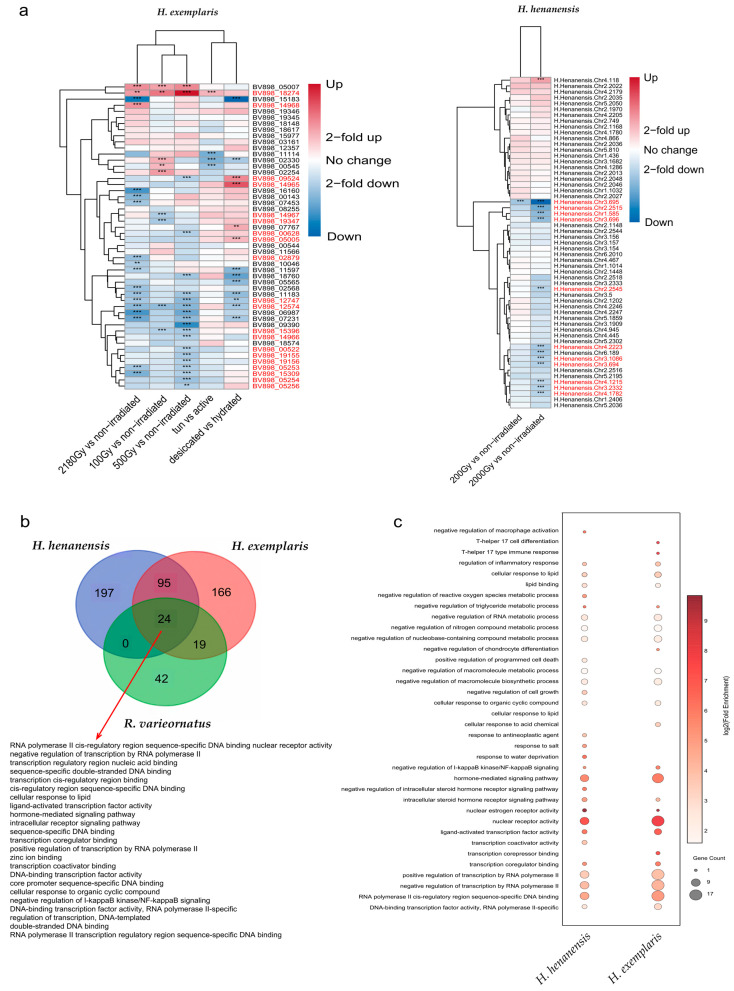
Differential expression and functional analysis of C4-type ZFP genes in tardigrades under extreme conditions. (**a**) Differential expression of C4-type ZFP genes of *H. Exemplaris* and *H. henanensis* in extreme environments; the blue colour in the heatmap represents downregulation, while the red colour represents upregulation, with significance only marked for |fold change| > 2 and *p* < 0.05 (** represents *p* < 0.01, *** represents *p* < 0.001), and the red molecular tag on the right represents the differentially expressed genes encoding C4-type ZFPs of Group 5. (**b**) Number of enriched GO terms in differentially expressed C4-type ZFP genes in Group 5 of *H. henanensis*, *H. exemplaris*, and *R. varieornatus*. (**c**) GO enrichment analysis of C4-type ZFP genes in Group 5 downregulated under radiation conditions in *H. henanensis* and *H. exemplaris.* The figure displays selected enrichment entries (q-value < 0.05, Fold Enrichment > 2).

C4-type ZFP genes differentially expressed under extreme conditions (radiation or desiccation) were identified in each tardigrade species. Among the 185 C4-type ZFP genes of tardigrades, 71 exhibited differential expression, 37 of which were identified in Group 5. The remaining differentially expressed genes were scattered across various other groups. The differentially expressed genes in Group 5 primarily originate from *H. henanensis*, *H. exemplaris*, and *R. varieornatus*. Further GO enrichment analysis was conducted on the differentially expressed genes of each of the three tardigrade species in Group 5, followed by an overlap analysis. The results revealed that 24 core GO terms were enriched in the differentially expressed genes of all three tardigrade species in Group 5, primarily involving DNA binding and transcriptional regulation, as well as key signal perception and transduction pathways, such as negative regulation of I-kappaB kinase/NF-kappaB signaling. Further analysis revealed that under irradiation conditions, all differentially expressed C4-type ZFP genes in *H. henanensis* from Group 5 were downregulated, and the differentially expressed C4-type ZFP genes in *H. exemplaris* from Group 5 were also primarily downregulated (Table S13, Figure 6a,b and Figure S9).

To elucidate the functional convergence and species-specific characteristics of the differentially expressed genes within Group 5 under radiation stress, Gene Ontology (GO) enrichment analysis was performed separately on the Group 5 members that were significantly downregulated in species *H. henanensis* and *H. exemplaris* following radiation exposure. Applying a stringent significance threshold (q-value < 0.05, Fold Enrichment > 2), we identified a set of significantly enriched biological processes and molecular functions. From the viewpoint of molecular function, the downregulated genes in both species were significantly enriched in terms related to transcriptional regulation. Most notably, nuclear receptor activity exhibited the highest enrichment significance and fold change. The highly related term ligand-activated transcription factor activity was also strongly enriched. Additionally, terms such as RNA polymerase II cis-regulatory region sequence-specific DNA binding, DNA-binding transcription factor activity, RNA polymerase II-specific, and transcription coregulator binding were highly enriched. In the biological process category, a key common finding was the significant enrichment of negative regulation of I-kappaB kinase/NF-kappaB signaling in both species. Concurrently, hormone-mediated signaling pathway and intracellular steroid hormone receptor signaling pathway were also significantly enriched. The downregulated genes were also broadly enriched for negative regulation terms targeting various biosynthetic and metabolic processes. These included negative regulation of macromolecule biosynthetic process, negative regulation of nucleobase-containing compound metabolic process, negative regulation of RNA metabolic process, and negative regulation of nitrogen compound metabolic process (Figure 6c).

## 3. Discussion

### 3.1. C4-Type ZFPs Are Known to Have Various Crucial Functions but Their Distribution and Roles in Tardigrades Remain Elusive

The C4-type zinc finger domain is widely present in the DNA-binding regions of nuclear hormone receptors. It typically comprises two zinc finger modules with significant sequence divergence that are difficult to directly align, along with a C-terminal extension. The first zinc finger module is responsible for recognising specific DNA sequences, while the second primarily participates in protein dimerisation [19]. This domain recognises and binds specific hormone response elements, subsequently mediating transcriptional activation or repression of target genes via its linked C-terminal ligand-binding domain [19,20]. Furthermore, the C4-type DNA-binding domain participates in multiple functions including nuclear localisation and interactions with transcription factors and co-activators. As a crucial class of transcriptional regulators, steroid/nuclear hormone receptors are extensively involved in physiological processes such as embryonic development, cell differentiation, and homeostasis maintenance, functioning in the nucleus as ligand-dependent dimers [32,33,34,35]. Consequently, C4-type ZFPs possess significant functions across diverse biological processes. In this study, statistical analysis revealed that C4-type zinc finger domains are exclusively present in the eukaryotes. This finding aligns with previous studies demonstrating that nuclear receptors are likewise restricted to metazoans (e.g., *A. queenslandica*, *C. elegans*, and *D. melanogaster* to *H. sapiens*) yet absent in protozoa, algae, fungi, or plants [36,37,38,39,40,41,42]. Furthermore, C4 type ZFPs have been studied in model organisms such as *Homo sapiens*, mice, and nematodes, as well as in numerous other animals [43]. A known family of transcription factors involved in tissue-specific gene regulation is the GATA family. All GATA proteins recognise the GATA consensus sequence and contain the C4 zinc finger motif. One member of this family, GATA-3, is considered a candidate regulatory gene potentially involved in T cell differentiation in humans and mice [44,45,46]. Over two decades ago, the sequencing of the *Caenorhabditis elegans* genome revealed unprecedented richness in the C4 zinc finger orphan nuclear hormone receptor family. These receptors are encoded by 267 distinct nhr genes. Analyses of over 100 nematode genomes suggest that the expansion of the nhr family represents an adaptation to complex sensory environments. NHR proteins may function as sensory receptors, detecting external or internal sensory cues to regulate the animal’s sensory responses to environmental stimuli and its internal metabolic state [47]. Research has also identified a novel protein in *Litopenaeus vannamei* shrimp, primarily localised in the cytoplasm with minor nuclear presence. This protein, containing a single C4-type zinc finger domain (SZnf), positively regulates the expression of multiple antimicrobial peptides both in vitro and in vivo, thereby participating in antimicrobial responses [48]. The foregoing demonstrates that C4-type zinc finger domain-containing proteins play crucial roles in T cell differentiation, environmental adaptation, and antimicrobial capabilities across other species. However, systematic investigations within tardigrades remain absent at present.

### 3.2. Phylogenetic and Conservation Analysis of C4-Type ZFPs in Tardigrades

Phylogenetic analysis of C4-type ZFPs and conservation studies of their motifs in tardigrades yielded the following principal conclusions: Firstly, the phylogenetic tree divides them into six groups. Group 5 predominantly contains tardigrade C4-type ZFPs. Motif analysis indicates that evolutionary relationships are directly reflected in motif conservation—intra-group members exhibit minimal variation, whereas inter-group members display substantial differences in motif type and position. Moreover, among all six conserved motifs, motif 1 and motif 2—crucial for DNA recognition and binding—exhibit high conservation across nearly all phylogenetic members, absent only in a handful of cases, underscoring their functional centrality. Notably, Group 5, as a distinct branch, may have undergone functional specialisation. A substantial number of its members incorporate not only motif 1 and motif 2 of the nuclear hormone receptor DNA-binding domain and the nuclear hormone receptor ligand-binding domains motif 3 and motif 5, but also the functionally uncharacterised motifs 4 and 6. This suggests that they may undertake unique protein–protein interactions, transcriptional regulation, or signal response functions. To clearly demonstrate the three-dimensional structures of all motif types, we selected a representative protein (evm.model.LG02.2515, the atrial natriuretic peptide receptor 1) from Group 5. We used AlphaFold for protein structure prediction and PyMOL to colour the positions of six motifs in the protein’s three-dimensional structure (Figure S10).

### 3.3. Prediction of Cis-Acting Elements for C4-Type ZFP Genes in Tardigrades

Predictions of cis-acting elements reveal that multiple regulatory sequences associated with stress responses are widely distributed across the tardigrade genome, encompassing stress signal integration, specific environmental stresses, pathogen immune responses, and auxiliary coregulation. Quantitatively, proteins harbouring the following motifs have been found to be more abundant in promoter regions (2000 bp upstream of the start codon) of C4-type ZFP genes within Group 5: fkh-2 [49,50,51], involved in the integration of growth factor signalling, oxidative stress, and inflammation; hsf-1 [52,53,54], a transcription factor that binds DNA and induces stress response; xbp-1 [55,56,57,58], key for maintaining endoplasmic reticulum homeostasis; lin-14 [59], known to promote nematode larval survival under high temperature; lin-48 [60], which responds to salt stress; and ceh-36 [61], responsible for sensing chemical stimuli. Collectively, elements associated with extreme environmental responses are widely present in tardigrades, such as motifs ‘[AG] TAAACA, GAAC [AG] C, and GGATTA’ corresponding to fkh-2, lin-14, and ceh-36. Analysis of differentially expressed transcriptome data revealed that 24 out of 185 C4-type ZFP genes exhibited upregulation under extreme environmental conditions. Furthermore, almost all of these upregulated genes contained at least one type of the aforementioned cis-acting elements (Table S7). It is speculated that the C4-type ZFP gene family may play a crucial role in tardigrade adaptation to extreme environments by regulating key physiological processes such as high-temperature survival, oxidative stress resistance, inflammation resistance, and other aspects. However, our analytical approach exhibits certain limitations. On the one hand, owing to the absence of a known database of tardigrades’ transcription factor binding motifs, and given that tardigrades and nematodes share a relatively closer evolutionary relationship compared to other classical model organisms, this analysis used nematode-derived transcription factor binding motifs to infer the presence of motifs within the upstream sequences of C4-type ZFP genes in tardigrades. Nevertheless, within the broader animal kingdom, nematodes and tardigrades are not particularly closely related evolutionarily. On the other hand, while our predictions indicate the presence of stress response-associated motifs in the promoter upstream region, this does not necessarily imply corresponding functional regulation. The results of differential expression analysis of transcriptomic data merely constitute supplementary evidence supporting our findings, which merely represent speculations regarding potential functional regulatory roles; whether these speculations hold true will require further in-depth research for verification in the future.

### 3.4. Duplication of C4-Type ZFP Genes in Tardigrades

From an evolutionary perspective, dispersed duplication constitutes the primary driving force behind the expansion of C4-type ZFP genes in the tardigrades *H. exemplaris* and *H. henanensis*, whilst tandem duplication and proximal duplication play significant auxiliary roles. Notably, tandem duplication not only propelled the amplification of this gene family in the three tardigrade species *H. exemplaris*, *H. henanensis* and *R. varieornatus*, but also played a pivotal role in the formation of the functionally specialised Group 5 branch. Functional enrichment analysis indicates that the genes replicated in tandem in Group 5 may enhance their transcriptional regulation ability, suppress high-energy-consuming processes such as reproduction and growth in extreme environments, and strengthen the maintenance of key organs and energy reserves, thereby improving survival resilience. Further Ka/Ks analysis revealed that the vast majority of homologous gene pairs underwent purifying selection in both intraspecific and interspecific comparisons, indicating highly conserved functions. Only a few gene pairs showed evidence of positive selection, suggesting potential for functional diversification. Chromosomal localisation visualisation in *H. exemplaris* and *H. henanensis* revealed similar patterns for C4-type ZFP genes: uneven distribution overall, yet localised clusters of duplicated genes, with these clustered regions exhibiting collinearity between the two species. Furthermore, a combined analysis of homologous genes and gene duplication event types revealed that, with the exception of a few genes, the majority of locally duplicated C4-type ZFP genes had already completed duplication prior to the divergence of the two species, representing an ancient event in the evolutionary history of tardigrades. The above findings indicate that the C4-type ZFP genes of these two tardigrades share similar evolutionary characteristics while also exhibiting certain selective variations.

### 3.5. Special Non-Nuclear Localisation for C4-Type ZFPs in Tardigrades

Both the subcellular localisation types of C4-type ZFPs and the number of non-nuclear-localised C4-type ZFPs in tardigrades are more abundant in Group 5 compared to other groups. In other species, there are also some non-nuclear-localised C4-type ZFPs. Among these members, there is a significant proportion coming from *C. elegans*. Furthermore, in *C. elegans*, the members of non-nuclear-localised C4-type ZFPs are mainly present in Group 2. The non-nuclear-localised C4-type ZFPs are primarily found in *C. elegans* and tardigrades as well as scattered among other species throughout the phylogenetic tree. Both *C. elegans* and tardigrades belong to the superphylum Panarthropoda [62], and previous studies indicate that *C. elegans* possesses the ability to enter a ‘dauer’ stage characterised by a significantly reduced metabolic rate to withstand adverse environments [63], resist changes in extracellular osmotic pressure [64,65,66,67], perceive and respond to shifts in growth temperature or harmful heat stress [68], and prolong their lifespan under low-dose ionising radiation by elevating persistent reactive oxygen species (ROS) and mitochondrial ROS levels [69]. These characteristics bear similarities to the cryptobiotic mechanisms and tolerance to desiccation and radiation observed in tardigrades. It is therefore hypothesised that the survival of tardigrades and *C*. *elegans* in extreme environments may be linked to the non-nuclear-localised C4-type ZFPs, which may exert specific functional roles either outside the cell nucleus or after being actively transported from the cytoplasm into the nucleus. Notably, these are based on bioanalyses and speculations. The determination of subcellular localisation is based on the combined predictive results from the TargetP-2.0 and DeepLoc 2.1 tools. All predicted positioning results lack experimental verification. Some positioning results may have false positives. Specifically, when using TargetP-2.0 for prediction, it was observed that two proteins (evm.model.LG02.2013 and evm.model.LG02.2036) possessed mitochondrial transfer peptides. However, when DeepLoc 2.1 was employed for prediction, these two molecules were indicated to localise in the nucleus. Given the challenge of excluding false positives in mitochondrial localisation, we cautiously selected the results from DeepLoc 2.1 as our final localisation outcomes. Previous studies indicate that nuclear receptors can be categorised into two major classes: cytoplasmic Type I nuclear receptors and nuclear Type II nuclear receptors. Small lipophilic molecules diffuse through the cell membrane and bind to these receptors, inducing conformational changes that trigger a cascade of downstream events. This process guides nuclear receptors to DNA transcription regulatory sites, ultimately leading to the upregulation or downregulation of gene expression [70]. Specifically, when ligands bind to Type I nuclear receptors, they induce heat shock protein dissociation and homodimerisation. These receptors are then actively transported from the cytoplasm into the nucleus, where they bind to specific DNA sequences known as hormone response elements (HREs). Subsequently, the nuclear receptor/DNA complex recruits additional proteins that transcribe the DNA downstream of HREs into messenger RNA, ultimately leading to protein synthesis and consequent alterations in cellular function [71]. According to our subcellular localisation prediction results, the C4-type ZFPs that not only localised in nuclei and may also be located in the cytoplasm. We speculate that those C4-type ZFPs located in ‘cytoplasm|nucleus’ may be classified as Type I nuclear receptors and promote survival in extreme environments for tardigrades and *C. elegans* by performing specialised functions after being actively transported from the cytoplasm into the nucleus.

### 3.6. The Role of C4-Type ZFP Genes of Group 5 in Extreme Environmental Response

Comparative GO enrichment analysis was conducted on the subset of C4-type ZFP genes from the phylogenetic Group 5 that were downregulated in response to radiation in two tardigrade species. In *H. henanensis*, all differentially expressed Group 5 members were downregulated, while in *H. exemplaris*, downregulation was the predominant trend. This parallel transcriptional response suggests a potential convergence in regulatory logic. Functional profiling reveals a core shared signature: the downregulated genes in both species are robustly enriched for ligand-activated transcription factor activity and nuclear receptor activity. This indicates that the Group 5 members affected by radiation stress in both species primarily constitute a network of nuclear receptor-type transcription factors. A key common finding is the significant enrichment of negative regulation of I-kappaB kinase/NF-κB signaling. Previous studies have shown that the NF-κB pathway is a classic pathway that plays a central role in various biological processes such as immune response, inflammation, cell survival, and proliferation [72,73,74]. Based on this pattern, we propose a testable hypothesis: that radiation-induced downregulation of this specific subset may lead to a potential derepression of the NF-κB pathway, which could be part of a conserved survival response. Furthermore, the concurrent downregulation of genes associated with broad repressive functions, such as negative regulation of macromolecule biosynthetic process, points to a large-scale reprioritization of cellular resources likely favouring damage repair over growth. Our inferences are derived from in silico comparisons of enrichment patterns between these two specific datasets. We acknowledge that differences in experimental design, radiation dosage, and biological context between the studies for *H. henanensis* and *H. exemplaris* preclude definitive claims regarding the strength or universality of the conservation. More importantly, the proposed biological roles—such as a network of nuclear receptor-type transcription factors—are predictive models generated from computational associations, not established mechanisms. These should be regarded as a prioritised working hypothesis for functional investigation. The nuances specific to each species in enriched terms, such as differential stress response themes, are interesting and merit further study; however, they do not, in themselves, constitute proof of distinct adaptive strategies. In summary, our analysis of two tardigrade species identifies a downregulated transcriptional module in irradiation (some C4-type ZFPs of Group 5) with a coherent functional signature centred on nuclear receptor-like regulation and NF-κB pathway interaction. This generates a specific and falsifiable predictive framework: that these genes act as a regulatory interface whose suppression under stress may facilitate survival signalling and resource reallocation. Future research must employ functional genetics—such as targeted knockdown/overexpression of key Group 5 genes in tractable models—to validate their predicted role in modulating NF-κB activity and stress resilience, thereby translating this correlative pattern into mechanistic understanding.

## 4. Materials and Methods

### 4.1. Identification of Zinc Finger Domains

The hmmscan programme from HMMER 3.4 [75] was used to perform domain annotation on protein sequences from four tardigrades and three major domains (E-value ≤ 1 × 10^−2^). The Pfam-A.hmm file used for searching and zinc finger domain information for statistical analysis were downloaded from the InterPro and Pfam databases (https://www.ebi.ac.uk/interpro/download/Pfam/, accessed on 23 May 2025, and http://pfam.xfam.org, accessed on 27 February 2025). The sources of the longest protein sequence file corresponding to each gene in tardigrade genomes are as follows: 14,701 sequences from *H. henanensis* were generated by our research group; 19,939 sequences from *H. exemplaris* and 20,465 sequences from *P. metropolitanus* were downloaded from the National Center for Biotechnology Information database (https://www.ncbi.nlm.nih.gov/, accessed on 18 March 2024); and 19,534 sequences for *R. varieornatus* were primarily downloaded from the National Center for Biotechnology Information database, incorporating protein sequences corresponding to 13 mitochondrial genes from the UniPortKB database (https://www.uniprot.org, accessed on 15 October 2024).

### 4.2. Identification of the C4-Type ZFP Gene Family and Prediction of Their Physicochemical Properties and Secondary Structures

The identification of the C4-type ZFP gene family in tardigrades was achieved through a comprehensive approach employing three methods: 1. BLAST databases were constructed using protein sequences corresponding to genes from four tardigrades. High-quality C4-type ZFP sequences from other species, manually annotated and downloaded from the UniPortKB database (https://www.uniprot.org, accessed on 19 March 2025), were combined with high-quality C4-type zinc finger domain seed sequences, manually screened and validated from the Pfam databases (https://www.ebi.ac.uk/interpro/download/Pfam/, accessed on 20 May 2025), to form query files. These were then subjected to BLASTp searches (E-value ≤ 1 × 10^−5^). 2. An HMM model was constructed based on known C4-type ZFP sequences. This model was used to search the protein sequences corresponding to genes from the four tardigrade species via the hmmsearch programme in HMMER 3.4. The HMM model construction process employed MAFFT v7.525 in default mode alongside the hmmbuild programme. The hmm search was conducted in three stages: a model was constructed from manually curated and validated high-quality C4-type zinc finger domain seed sequences downloaded from the Pfam database (E-value < 1 × 10^−2^), and a model was constructed from all sequences associated with C4-type zinc finger domains downloaded from the Pfam databases (https://www.ebi.ac.uk/interpro/download/Pfam/, accessed on 20 May 2025) (E-value < 1 × 10^−5^). 3. Based on the C4-type zinc finger domain HMM model downloaded from the Pfam databases (https://www.ebi.ac.uk/interpro/download/Pfam/, accessed on 20 May 2025), perform searches using the hmmsearch programme on protein sequences corresponding to genes from four tardigrade species (E-value < 1 × 10^−2^). Merge the results identified by the above three methods, remove redundancy, and use the consolidated set as candidate proteins for validation of conserved domains. Conserved domain validation was conducted against the NCBI-CDD database (https://www.ncbi.nlm.nih.gov/cdd/, accessed on 31 March 2025), SMART database (http://smart.embl-heidelberg.de/, accessed on 31 March 2025), Pfam database (http://pfam.xfam.org/search#searchBatchBlock, accessed on 1 April 2025), and InterPro database (accessed on 1 April 2025). The validation results were merged, and candidate proteins lacking the C4-type domain were manually removed. The physicochemical properties and secondary structure predictions of the filtered C4-type ZFPs were performed using online platforms (https://web.expasy.org/protparam/ and https://npsa-prabi.ibcp.fr/cgi-bin/secpred_gor4.pl, accessed on 3 April 2025) [76,77].

### 4.3. Phylogenetic Clustering

Sequence alignment of C4-type ZFPs from tardigrades and other species was performed using the --localpair mode of MAFFT v7.525 [78], and the sequences were subsequently trimmed using trimAl v1.5.rev0 [79]. The trimmed sequences were then subjected to phylogenetic tree construction via FastTree Version 2.1.11 in Lg+Gamma mode [80]. The resulting Newick-format phylogenetic tree file was uploaded to iTOL (https://itol.embl.de, accessed on 11 June 2025) for visual refinement [81].

### 4.4. Analysis of Conserved Motifs

Identify conserved motifs within the C4-type ZFPs of four tardigrades using the online software MEME Suite 5.5.9 (https://meme-suite.org/tools/meme, accessed on 20 November 2025), with the maximum value set to 6 and other parameters set to default values [82]. The online database InterPro 107.0 (https://www.ebi.ac.uk/interpro/, accessed on 20 November 2025) was consulted to determine the functional significance of the six motif sequences. Visualisation of the conserved motifs was performed using TBtools-II v2.227 software [83]. The three-dimensional structures of all motif types in a representative molecule were demonstrated using both AlphaFold [84] (https://deepmind.google/science/alphafold/, accessed on 21 January 2026) and PyMOL 3.1 [85].

### 4.5. Prediction of Cis-Acting Elements

To investigate regulatory elements associated with stress resistance in the promoter regions of C4-type ZFP genes across four tardigrade genomes, tools including gffread v0.11.7, samtools 1.9, and bedtools v2.27.1 [86,87,88,89,90] were employed based on genome files and genome annotation files (GFF format). The 2000 bp sequence upstream of the C4-type ZFP gene start codon ATG was extracted. Subsequently, information on the closely related nematode species was downloaded from the CORE dataset within the JASPAR2024 database (https://jaspar.elixir.no, accessed on 21 September 2025). The position frequency matrix file was converted into regular expressions for prediction. The regular expression conversion relies on a Python 3.8 environment. First, the Biopython motifs.parse() function read JASPAR-formatted data, utilising the degenerate_consensus attribute to generate IUPAC degenerate consensus sequences. During the pattern conversion stage, a custom iupac_to_regex_pattern() function implemented complete mapping from IUPAC codes to regular expressions, encompassing all standard degenerate bases. In the quality control phase, the validate_regex_patterns() function combined with re.compile() performed rigorous syntax validation on all generated regular expressions, ensuring accuracy and reliability for subsequent sequence scanning. Sequence scanning prediction and visualisation rely on R version 4.2.1. First, the readDNAStringSet function from the Biostrings package is used to read sequence files. Subsequently, based on cis-element motifs derived from nematodes, the customised predict_elements_count function combined with the gregexpr function performs regular expression matching scans to count the occurrence frequency of each element within the sequences. Finally, visualisation is achieved using the ggplot2 package.

### 4.6. Analysis of Duplication of C4-Type ZFP Genes

To identify replication events of C4-type ZFP genes in tardigrades, the NCBI BLAST+ software suite (version 2.17.0+) was obtained from the official NCBI FTP server (https://blast.ncbi.nlm.nih.gov/Blast.cgi, accessed on 29 July 2025) and installed for homologous sequence alignment. Subsequently, the blastp programme was employed to align the target proteome against itself, with an E-value threshold of <1 × 10^−5^ set to ensure statistical significance in homological relationship identification. Results were output in tabular format. Finally, the aforementioned alignment result files were submitted as input to the duplicate_gene_classifier programme within the MCScanX software package (v1.1.9). This enabled automated classification and identification of different types of duplicate genes, such as segmental duplications and tandem duplications. The MCScanX software package was downloaded from GitHub (https://github.com/wyp1125/MCScanX.git, accessed on 29 July 2025) and deployed locally [91,92,93]. GO enrichment analysis for tandem-duplicated genes was performed based on a hypergeometric distribution model. *p*-values were corrected using the Benjamini–Hochberg algorithm, with ±lg(P) representing enrichment or deficiency, respectively—i.e., enrichment yields positive values while deficiency yields negative values.

### 4.7. Identification of Gene Age

The method used for determination of gene age of the four tardigrades is the same as that used in our previous studies [29]. Briefly, we conducted reciprocal sequence alignment of all protein sequences of each tardigrade species with the protein sequences of the 3376 representative species. Matches with both E-values less than 1 × 10^−5^ were regarded as homologous genes. The number of homologous genes in each species for each gene was calculated and the evolutionary age of each gene was inferred based on the phylostratigraphy approach.

### 4.8. Analysis of Ka/Ks Ratio

Utilising positional information from the genome annotation file (GFF format), the gffread v0.12.8 tool was employed to extract the coding sequences (CDSs) of C4-type ZFP genes from the genome file. Subsequently, multiple sequence alignment was performed using the ClustalW (Codons) method within MEGA11 [94], with parameters set to default values. Following generation of the .mas file, pairwise distances were calculated with a Bootstrap repetition value of 1000. The substitution model employed the Nei–Gojobori method (Jukes–Cantor), with substitution types set to n, Nonsynonymous only, and s, Synonymous only. The Ka and Ks values were calculated for homologous gene pairs. Finally, a Python script was utilised for batch computation to derive the Ka/Ks ratios for homologous gene pairs.

### 4.9. Chromosomal Localisation

Chromosomal localisation for C4-type ZFP genes was visualised using the online tool MG2C v2.1 (http://mg2c.iask.in, accessed on 5 August 2025) [95].

### 4.10. Prediction of Subcellular Location

Subcellular localisation predictions for C4-type ZFPs in four tardigrade species and other organisms were performed using the online tools TargetP-2.0 (https://services.healthtech.dtu.dk/services/TargetP-2.0/, accessed on 18 January 2026) and DeepLoc 2.1(https://services.healthtech.dtu.dk/services/DeepLoc-2.1/, accessed on 18 January 2026). The prediction results of DeepLoc-2.1 are the main results, and the prediction results of Target P-2.0 are used as auxiliary verification. Data processing and visualisation were accomplished within a Python3.13 environment, utilising the pandas and matplotlib libraries [96].

### 4.11. Differential Expression Analysis of Transcriptome Data

Raw transcriptome sequencing data for *R. varieornatus*, *H. exemplaris*, and *P. metropolitanus* under extreme environmental conditions were downloaded from the European Nucleotide Database (https://www.ebi.ac.uk/ena/browser/home, accessed on 12 January 2025–10 February 2025). Differential expression analysis was performed using FastQC v0.12.1 + trimmomatic v0.39 + hisat2-align -s v2.2.1 + featureCounts v2.0.6 + DESeq2 v1.44.0 [97,98,99,100,101]. Genome sequence files and annotated gene annotation files (GTF format) for differential expression analysis were downloaded from the NCBI Genome Database (https://www.ncbi.nlm.nih.gov/datasets/genome/, accessed on 11 January 2025). Transcriptome data for *H. henanensis* under irradiation conditions were generated in our laboratory. Heatmaps depicting differential expression analysis results for C4-type ZFP genes were generated using the pheatmap package within the R environment.

### 4.12. Functional Enrichment Analysis

The genome GO annotations for four tardigrade species were obtained by merging and deduplicating results from two tools: InterProScan (version 5.73-104.0) and eggNOG-mapper (version 2.1.13) [102,103]. When processing with InterProScan, the parameters used were ‘-iprlookup -goterms -dp’. For eggNOG-mapper, the eggNOG DB v5.0.2 database and the parameter ‘-m diamond’ were employed. Ultimately, 10,127, 11,948, 12,196, and 13,911 genes were annotated with GO terms in *H. henanensis*, *R. varieornatus*, *H. exemplaris*, and *P. metropolitanus*, respectively. GO enrichment analysis of differentially expressed genes in Group 5 was similarly performed using a hypergeometric distribution model. *p*-values were corrected via the Benjamini–Hochberg method, with ±lg(P) representing enrichment or depletion, respectively (positive values for enrichment, negative for depletion). The figure displays only selected enrichment entries (q-value < 0.05, Fold_enrichment > 2). Visualisation was performed using the matplotlib and pandas libraries within the Python environment.

## 5. Conclusions

In summary, this study first systematically analysed the distribution characteristics of zinc finger domains across the three superkingdoms of life and four tardigrade species, selecting C4-type zinc finger domains as the subject for in-depth investigation. For the first time, by integrating comparative genomics, transcriptomics, and evolutionary analysis, we systematically elucidated the core regulatory mechanisms of C4-type ZFPs in tardigrade adaptation to extreme environments (radiation or desiccation). Key breakthroughs include: (1) revealing a unique Group 5 branch within the phylogenetic tree, whose gene promoter regions are highly enriched with multiple stress response elements—furthermore, this branch achieves functional specialisation through tandem duplication and may enhance survival by strengthening transcriptional regulation to withstand adverse environments; (2) elucidating pervasive purifying selection pressures and other similar evolutionary traits within this family; (3) a subset of C4-type ZFP genes, particularly within Group 5, was found to respond to extreme stresses such as radiation and desiccation. In both H. henanensis and H. exemplaris, almost all differentially expressed genes in Group 5 showed consistent downregulation under radiation exposure. Functional enrichment analysis indicated that these genes are associated with nuclear receptor transcription factor activity and are significantly linked to the negative regulation of NF-κB signalling, which could potentially derepress the NF-κB pathway while suppressing growth-related processes, thereby coordinating a shift in cellular resources toward damage repair. However, this interpretation remains a working hypothesis derived from computational evidence and requires validation in the future. This study provides valuable data resources and clues for further in-depth research on the function and mechanism of C4-type ZFPs in tardigrades.

## Data Availability

All data generated or analysed in this study are included in the Supplementary Materials. Transcriptome raw data may be downloaded from the European Nucleotide Database (https://www.ebi.ac.uk/ena/browser/home accessed on 12 January 2025–10 February 2025) according to the Run Accession specified in Table S3 or from the corresponding references.

## Supplementary Materials

The following supporting information can be downloaded at: https://www.mdpi.com/article/10.3390/ijms27041739/s1.
