# Supplementary material for: Characteristics of C4-Type Zinc Finger Proteins in Tardigrades and Their Responses in Extreme Environments
Source: Int J Mol Sci. 2026 Feb 11;27(4):1739. doi: 10.3390/ijms27041739 (PMC12941274; doi:10.3390/ijms27041739)
Supplement: Supplementary file 1 [file ijms-27-01739-s001.zip › Caption of Supplementary Figures and Tables.pdf]

Supplementary Figure S1. Distribution of major stress and hormone response related cis-acting regulatory elements in the promoters of *R. varieornatus*'s C4-type ZFP genes.

Supplementary Figure S2. Distribution of major stress and hormone response related cis-acting regulatory elements in the promoters of *H. exemplaris*'s C4-type ZFP genes.

Supplementary Figure S3. Distribution of major stress and hormone response related cis-acting regulatory elements in the promoters of *P. metropolitanus*'s C4-type ZFP genes.

Supplementary Figure S4. Distribution of C4-type ZFP genes duplication modes in *R. varieornatus* and *P. metropolitanus*.

Supplementary Figure S5. Quantitative Analysis on the Subcellular Localization Types of C4-type ZFPs in *H. exemplaris*

Supplementary Figure S6. Quantitative Analysis on the Subcellular Localization Types of C4-type ZFPs in *R. varieornatus*.

Supplementary Figure S7. Quantitative Analysis on the Subcellular Localization Types of C4-type ZFPs in *P. metropolitanus*.

Supplementary Figure S8. Quantitative Analysis on the Subcellular Localization Types of C4-type ZFPs in other species.

Supplementary Figure S9. Differential expression of C4-type ZFP genes of *R. varieornatus* and *P.metropolitanus* in extreme environments.

Supplementary Figure S10. 3D structure display of six motifs in a molecule (evm.model.LG02.2515) of Group 5.

Supplementary Table S1. The overall distribution of zinc finger domains across four tardigrade species and three superkingdoms.

Supplementary Table S2. The count of zinc finger domains in four tardigrade species and three superkingdoms and relative ratio.

Supplementary Table S3.1. Sources of raw transcriptome sequencing data and for three tardigrade species under extreme environmental conditions.
Supplementary Table S3.2. Differential expression results of *R. varieornatus* under radiation and desiccation conditions, as compared to its physiological state.
Supplementary Table S3.3. Differential expression results of *H. exemplaris* under radiation and desiccation conditions, as compared to its physiological state.
Supplementary Table S3.4. Differential expression results of *P. metropolitanus* under desiccation conditions, as compared to its physiological state.

Supplementary Table S4. The types among the twelve zinc finger domains involved in the differentially expressed molecules of three tardigrade species under extreme environmental conditions.

Supplementary Table S5. Physicochemical properties, secondary structure and phylogenetic tree groups of C4-type ZFP genes in four tardigrade species.

Supplementary Table S6. Distribution of conservative motifs in C4-type ZFPs of four tardigrade species.

Supplementary Table S7. Prediction of cis-acting elements of C4-type ZFP genes in four tardigrade species.

Supplementary Table S8. Enrichment and depletion analysis of C4-type ZFP gene tandem duplication events in tardigrade genomes.

Supplementary Table S9.1. Gene age of C4-type ZFP genes and phylogenetic tree groups in four tardigrade species.
Supplementary Table S9.2. Number of C4-type ZFP genes at different gene age levels in four tardigrade species.
Supplementary Table S9.3. Number of C4-type ZFP genes across different gene age levels of tardigrades in each group.

Supplementary Table S10. Ka/Ks analysis of C4-type ZFP genes in tardigrades.

Supplementary Table S11.1. The tandem duplication and proximal duplication C4-type ZFP genes of *H. henanensis* and its corresponding homologous genes in its own and *H. exemplaris* genomes.
Supplementary Table S11.2. The tandem duplication and proximal duplication C4-type ZFP genes of *H. exemplaris* and its corresponding homologous genes in its own and *H. henanensis* genomes.

Supplementary Table S12. Subcellular localization and phylogenetic grouping of C4-type ZFPs in four tardigrade species and other organisms.

Supplementary Table S13. Differentially expressed genes of C4-type ZFP in tardigrades under radiation and desiccation conditions.
