# Supplementary material for: Characteristics of C4-Type Zinc Finger Proteins in Tardigrades and Their Responses in Extreme Environments
Source: Int J Mol Sci. 2026 Feb 11;27(4):1739. doi: 10.3390/ijms27041739 (PMC12941274; doi:10.3390/ijms27041739)
Supplement: Supplementary file 1 [file ijms-27-01739-s001.zip › Figures S1-S10.pdf]

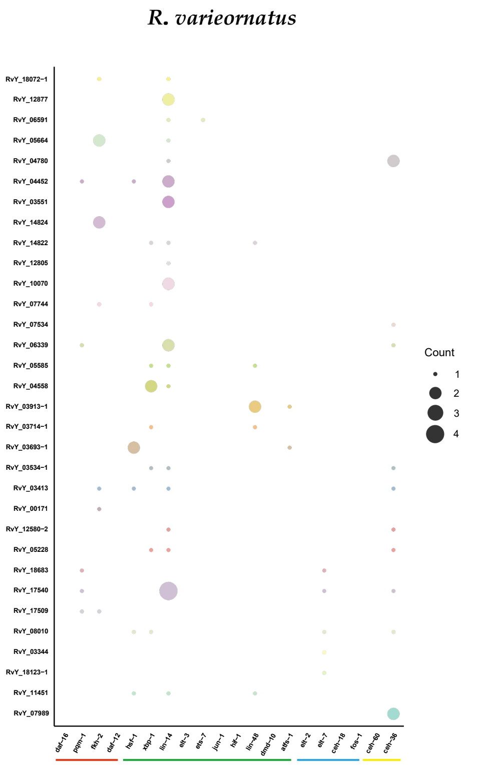

Figure S1. Distribution of major stress and hormone response-related cis-acting regulatory elements in the promoters of *R. varieornatus*'s C4-type ZFP genes.

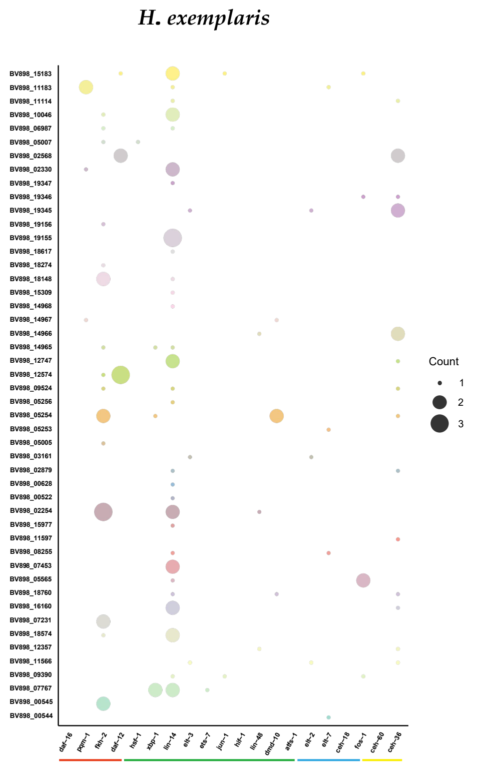

Figure S2. Distribution of major stress and hormone response-related cis-acting regulatory elements in the promoters of *H. exemplaris*'s C4-type ZFP genes.

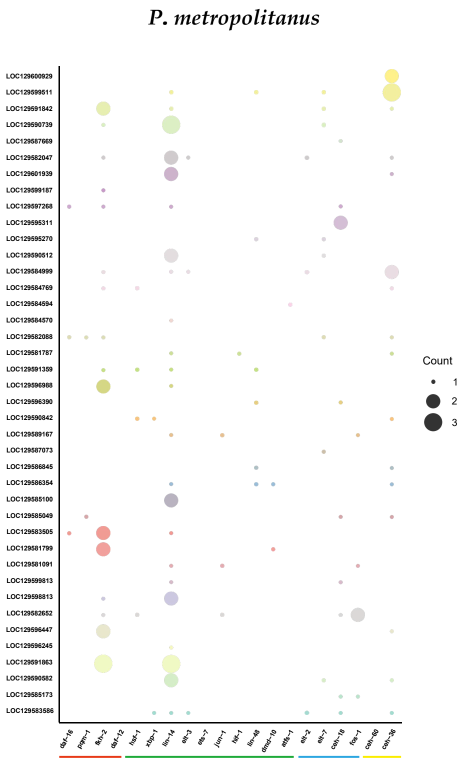

Figure S3. Distribution of major stress and hormone response-related cis-acting regulatory elements in the promoters of *P. metropolitanus*'s C4-type ZFP genes.

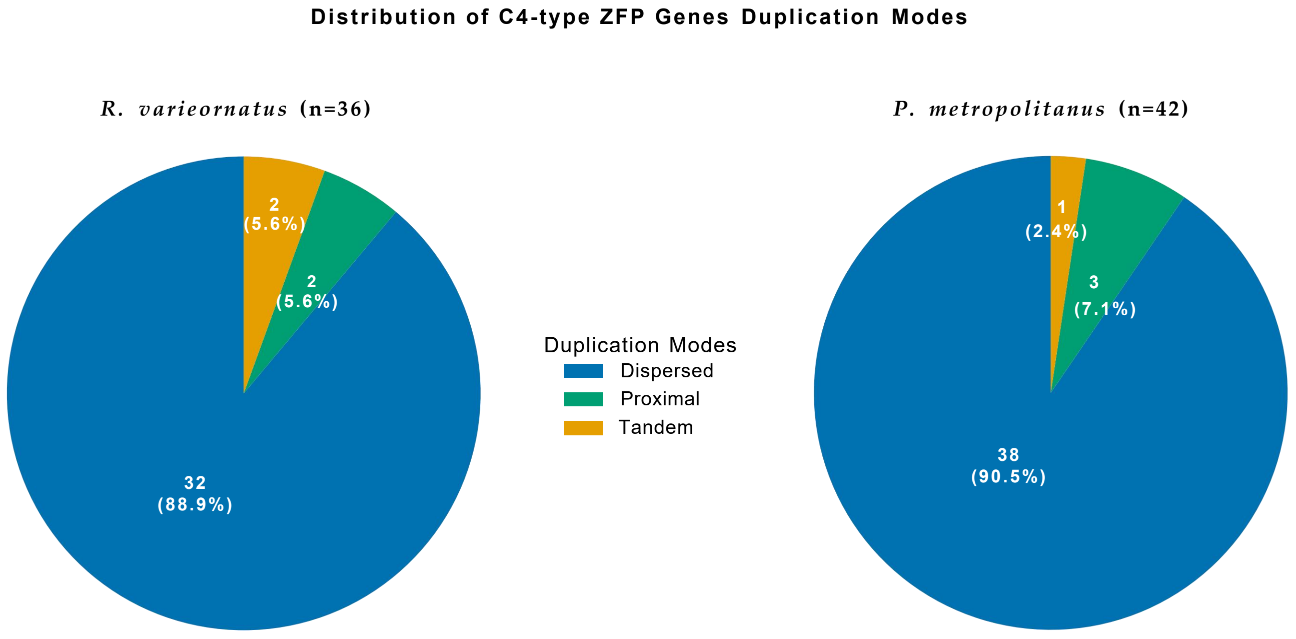

Figure S4. Distribution of C4-type ZFP genes duplication modes in *R. varieornatus* and *P. metropolitanus*, different genes duplication modes are represented by different colors, the white numbers represent the corresponding number and proportion of C4-type genes.

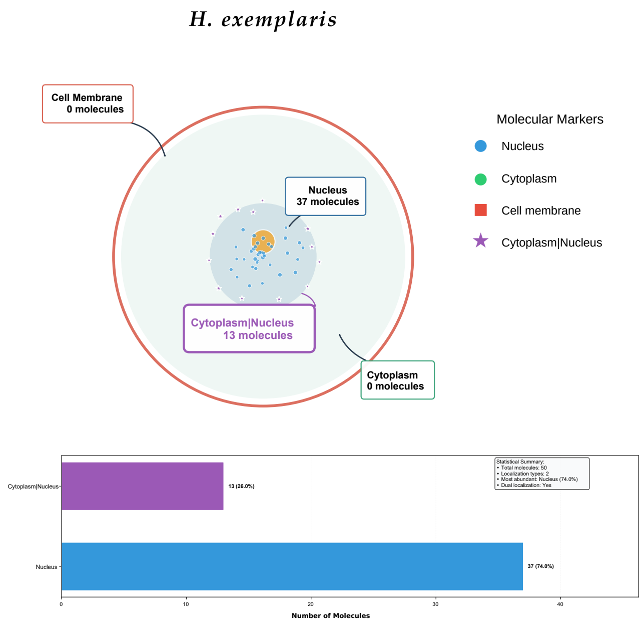

Figure S5. Quantitative Analysis on the Subcellular Localization Types of C4-type ZFPs in *H. exemplaris*.

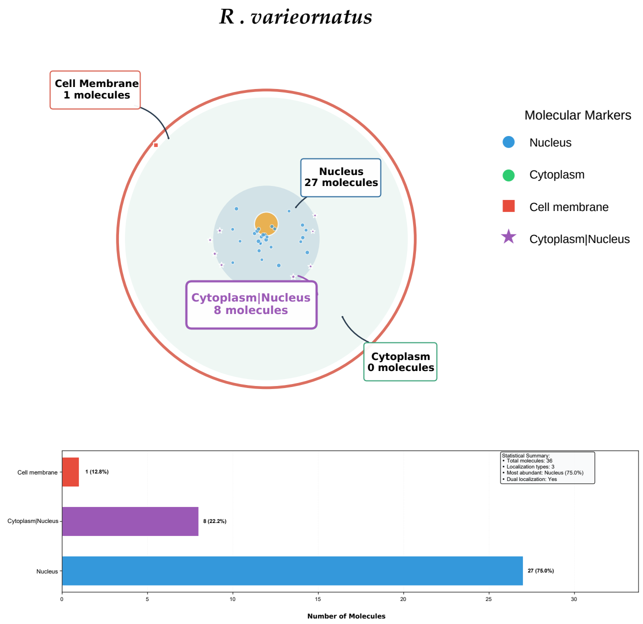

Figure S6. Quantitative Analysis on the Subcellular Localization Types of C4-type ZFPs in *R. varieornatus*.

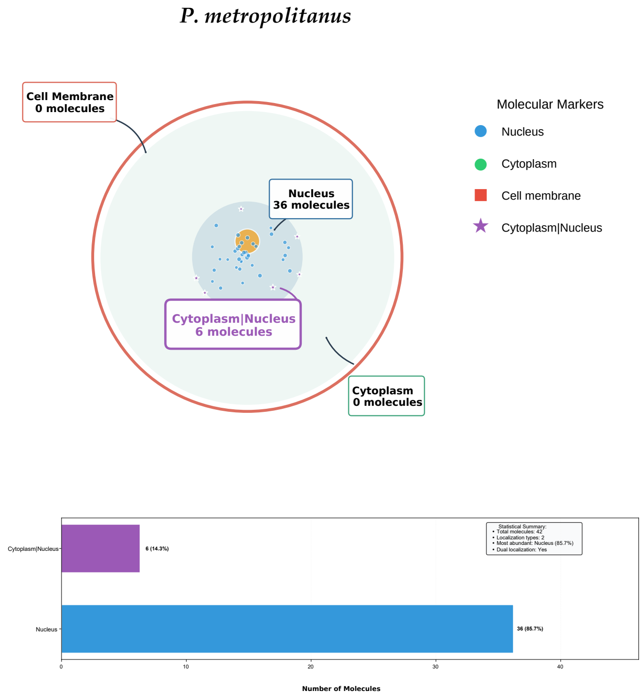

Figure S7. Quantitative Analysis on the Subcellular Localization Types of C4-type ZFPs in *P. metropolitanus*.

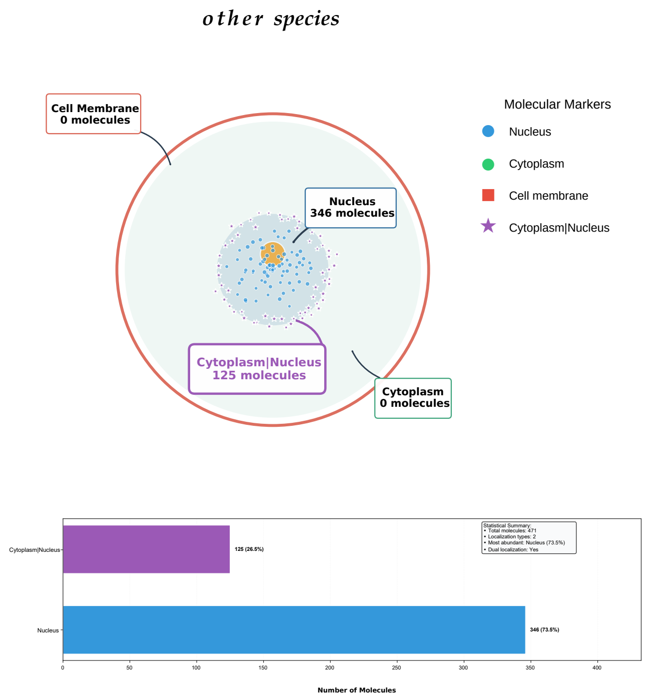

Figure S8. Quantitative Analysis on the Subcellular Localization Types of C4-type ZFPs in other species.

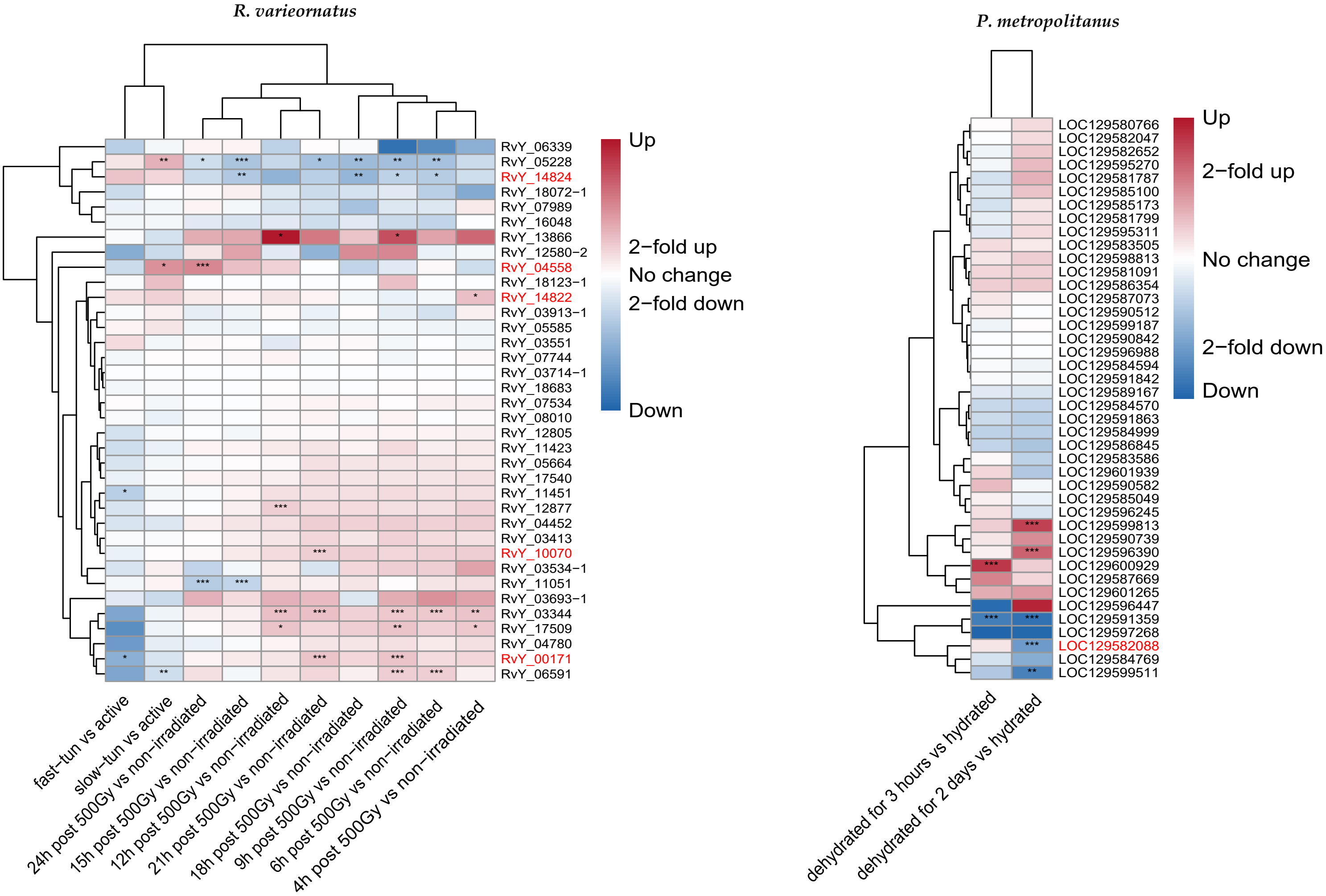

Figure S9. Differential expression of C4-type ZFP genes of *R. varieornatus* and *P. metropolitanus* in extreme environments, the blue color in the heatmap represents downregulation of expression, while the red color represents upregulation of expression, significance only marked for ∣Fold Change∣ > 2 and p < 0.05 (* represent p < 0.05, ** represent p < 0.01, *** represent p < 0.001) as well, the red molecular tag on the right represents the differentially expressed genes encoding C4-type ZFPs of Group 5.

evm.model.LG02.2515

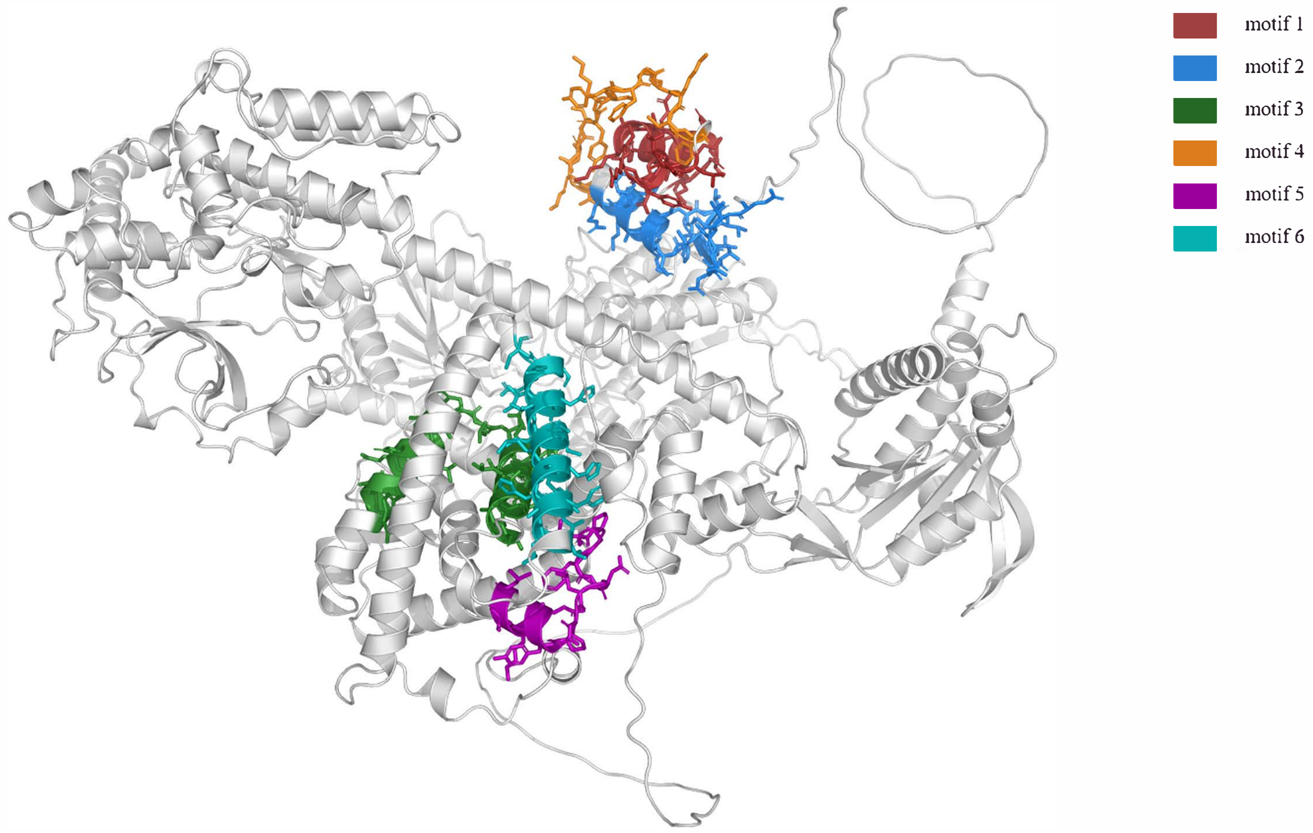

Figure S10. 3D structure display of six motifs in a molecule (evm.model.LG02.2515) of Group 5. The positions of motifs is predicted by MEME Suite 5.5.9 . Motif 1 (RTRNRCQACRFQKCLEVGMSL) and motif 2 (GDKATGVHYGVLTCEGCKGFFKRS) represent the nuclear hormone receptor DNA-binding domain. Motif 3 (YEFCRKLNDJGLDQTEYALLLAIVLF) and motif 5 (PGFSELPQEDQLALL) represent the nuclear hormone receptor ligand-binding domain. And the functions of motif 4 (KNLVYKCYFGGNCEI) and motif 6 (LQLJHRHYLDVLLDLLKQRCS) are unkonwn.
